# Method for the Detection of the Cleaved Form of Shiga Toxin 2a Added to Normal Human Serum

**DOI:** 10.3390/toxins13020094

**Published:** 2021-01-26

**Authors:** Lucrezia Rocchetti, Beatrice Munari, Elisa Varrone, Elisa Porcellini, Dorothea Orth-Höller, Reinhard Würzner, Domenica Carnicelli, Maurizio Brigotti

**Affiliations:** 1 Department of Experimental, Diagnostic and Specialty Medicine, University of Bologna, Via San Giacomo 14, 40126 Bologna, Italy; lucrezia.rocchetti@studio.unibo.it (L.R.); beatrice.munari2@unibo.it (B.M.); elisa.varrone2@unibo.it (E.V.); elisa.porcellini3@unibo.it (E.P.); domenica.carnicelli@unibo.it (D.C.); 2 Institute of Hygiene and Medical Microbiology, Medical University of Innsbruck, A-6020 Innsbruck, Austria; dorothea.orth@i-med.ac.at (D.O.-H.); reinhard.wuerzner@i-med.ac.at (R.W.)

**Keywords:** hemolytic uremic syndrome, cleaved Shiga toxin 2a, Shiga toxin-producing *Escherichia coli*

## Abstract

The pathogenesis of *Escherichia coli*-induced hemolytic uremic syndrome (eHUS) caused by infections with pathogenic Shiga toxin (Stx) producing *E. coli* (STEC) is centered on bacterial (e.g., Stx) and host factors (circulating cells, complement system, serum proteins) whose interaction is crucial for the immediate outcome and for the development of this life-threatening sequela. Stx2a, associated to circulating cells (early toxemia) or extracellular vesicles (late toxemia) in blood, is considered the main pathogenic factor in the development of eHUS. Recently, it was found that the functional properties of Stx2a (binding to circulating cells and complement components) change according to modifications of the structure of the toxin, i.e., after a single cleavage of the A subunit resulting in two fragments, A1 and A2, linked by a disulfide bridge. Herein, we describe a method to be used for the detection of the cleaved form of Stx2a in the serum of STEC-infected or eHUS patients. The method is based on the detection of the boosted inhibitory activity of the cleaved toxin, upon treatment with reducing agents, on a rabbit cell-free translation system reconstituted with human ribosomes. The method overcomes the technical problem caused by the presence of inhibitors of translation in human serum that have been stalled by the addition of RNAase blockers and by treatment with immobilized protein G. This method, allowing the detection of Stx2a at concentrations similar to those found by ELISA in the blood of STEC-infected patients, could be a useful tool to study the contribution of the cleaved form of Stx2a in the pathogenesis of eHUS.

## 1. Introduction

Renal thrombotic microangiopathy is the endpoint of the pathogenesis of *Escherichia coli*-induced hemolytic uremic syndrome (eHUS) in about 5–10% of children infected with Shiga toxin-producing *E. coli* strains (STEC) [1,2,3]. Circulating Shiga toxins (Stxs), in particular Stx2 type, are the main bacterial factors among those acting in concert in inducing eHUS [1,2]. During early toxemia and before the onset of eHUS, the toxins have been found free in sera or bound to circulating cells and then released as particulate toxins associated with 1 µm-sized extracellular vesicles [4]. These vesicles can attack target cells (endothelial cells in the kidney and brain, and other renal cells) by delivering Stxs together with other pathogenic factors which concur to eHUS pathogenesis, such as tissue factor and/or activated complement components [5,6,7]. The Stx2a subtype has been described to be involved by itself in triggering the activation of complement system [8,9]. Therefore, these multiple interactions between host factors and bacterial pathogenic factors are crucial in the development of eHUS. Many investigations have been undertaken to find out the relative contribution to eHUS pathogenesis of the different forms of Stx2a present in blood by considering this toxin subtype as a stable unchangeable element produced by pathogenic bacterial strains. Indeed, this toxin was subjected to intense scrutiny to unravel the AB5 structure, the sequence and folding, the specific glycolipid receptor (globotriaosylceramide, Gb3Cer), the intracellular routing and the enzymatic mechanism of action on ribosomes leading to irreversible halting of protein synthesis [1,2]. In particular, Stx2a holds a trypsin-sensitive region (Arg-X-X-Arg) in its A chain which is recognized by furin, the specific cellular protease responsible for the cleavage of the toxin during intracellular transport [10]. Furin-induced cleavage leads to the formation of two fragments, A1 and A2, linked by a disulfide bond which needs to be reduced to release the enzymatically active A1 fragment from the cleaved toxin [11]. This in vivo efficient process is mimicked by treatment of Stx2a with trypsin and dithiothreitol (DTT) in vitro. It is worth noting that extracellular cleavage of Stxs by proteases released by bacteria and/or present in the intestinal milieu has been described [12,13]. It is not known if blood proteases can also cleave Stxs in patients during the toxemic phase.

We have recently shown that the structure of the A chain of Stx2a is mandatory for its behavior in several steps of experimental eHUS pathogenesis [12]. Cleaved Stx2a having the A1 and A2 fragments bridged by the intact disulphide bond did not bind to human neutrophils through Toll-like receptor 4 (TLR4), and to the same receptor on monocytes and platelets, whereas it is fully active in binding to complement factor H [12]. Uncleaved toxin showed the opposite features [12]. It should be noted that when Gb3Cer-expressing human cells have been challenged with cleaved and uncleaved Stx2, the cytotoxicity profiles and the effects on cellular translation were very similar [12]. Despite this, the different effects of the two toxin forms on the pathogenetic steps preceding target cell intoxication might be crucial for eHUS development. It remains to be established what form of Stx2a is circulating in the blood of STEC-infected patients and, in particular, of the small group that develops eHUS. The outcome of STEC infections might be influenced not only by the array of pathogenic factors produced by a given STEC strain, or by the specific toxin subtype released by STEC, but also by the percentages of cleaved and uncleaved forms of the toxins circulating in patients’ blood.

Herein, a method is described to measure the enzymatic activity of the cleaved and uncleaved forms of Stx2a added to normal human sera by recording the inhibition of protein synthesis induced by the toxins in a cell-free luminometric rabbit reticulocyte-derived translation system set-up with human ribosomes. 

## 2. Results

### 2.1. Choice of the Cell-Free Translation System

The mandatory requirement for A chain cleavage and reduction to boost the enzymatic activity of Stxs was demonstrated 40 years ago [14] by measuring their inhibitory activity on the well-known cell-free radioactive translation system from rabbit reticulocyte lysate. In this system, native uncleaved Stx2a purified in our laboratory showed an IC_50_ (concentration of inhibitor causing 50% inhibition of protein synthesis) equal to 175 nM. We have recently developed a rabbit reticulocyte-derived fractionated system with human ribosomes translating exogenous synthetic mRNA coding for luciferases (luminometric assay) [15], which is ~50 times more sensitive to Stx2a (IC_50_ = 3.68 nM).

### 2.2. Choice of Reducing Conditions that Boost the Activity of Cleaved Stx2a in the Cell-Free Translation System

**Figure 1:**
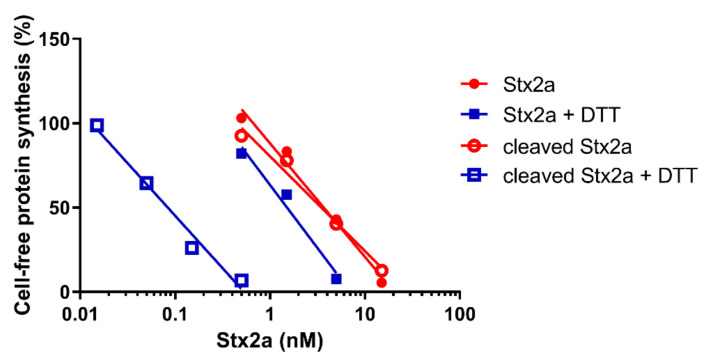
Effect of Stx2a on cell-free protein synthesis under reducing and non-reducing conditions. The IC_50_ values calculated from the straight lines depicted above are reported in Table 1.

**Figure 2:**
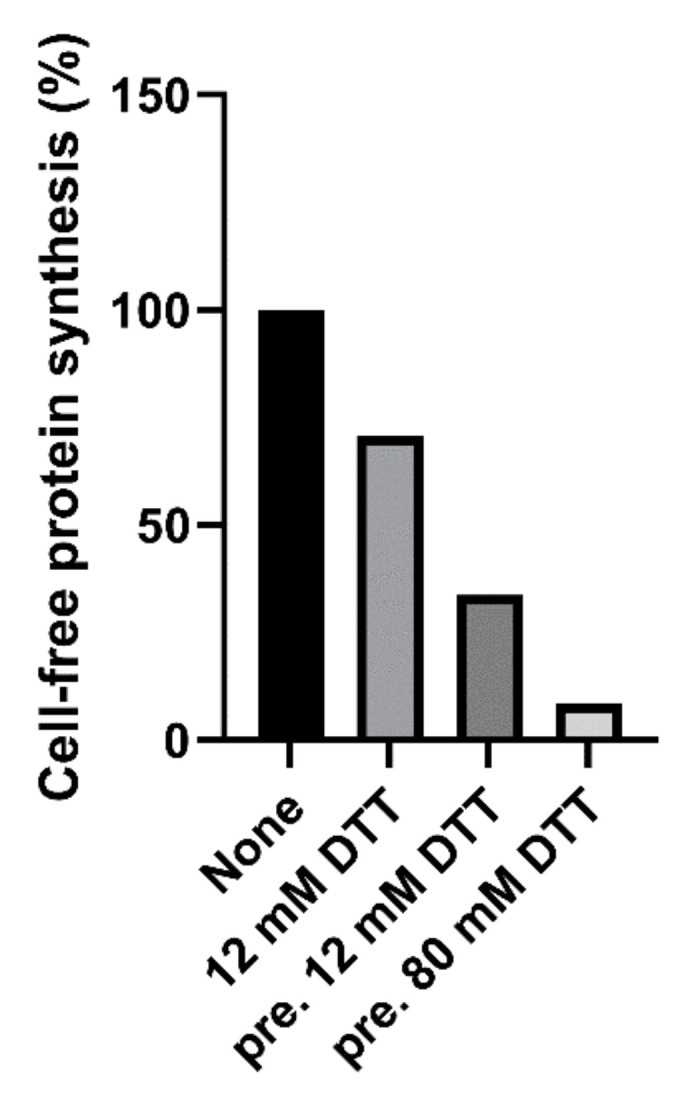
Effect of cleaved Stx2a (0.15 nM) on cell-free protein synthesis in the presence of 12 mM DTT, or after pretreatment (pre.) with 12 or 80 mM DTT.

Native and trypsin-treated Stx2a (cleaved Stx2a) assayed in this system (luminometric assay, Figure 1, red circles) showed similar inhibitory activities (Table 1), hence confirming that nicking of the Stx2a A chain insufficiently boosts toxin activity if the disulphide bond connecting the resulting A1 and A2 fragments is not reduced. To discriminate between cleaved and uncleaved forms of Stx2a, we added DTT to the system to obtain the release of the enzymatically active A1 fragment in cleaved toxin only. The DTT concentrations assayed were as in previously published methods (12–80 mM) [14,16] and the reducing agent was added directly to the system or in a pre-incubation step with the toxin. The best reducing conditions (2 µL of cleaved Stx2a preincubated for 15 min at 30 °C with 80 mM DTT in 3 µL final volume) was chosen according to Figure 2.

### 2.3. Determination of the Cleaved Form in Samples of Purified Stx2a

After the reducing treatment (Figure 1, blue squares. Table 1), the IC_50_ of uncleaved Stx2a only slightly changed (~2.5 fold, y-intercept difference: *p* < 0.01), whereas the IC_50_ of cleaved Stx2a was strikingly reduced (~42 fold, y-intercept difference: *p* < 0.0001). Therefore, it is possible to calculate the percentage of cleaved toxin in an unknown toxin preparation by calculating the fold-decrease of the IC_50_ under reducing with respect to non-reducing conditions. A simple flow chart allowing the calculation was set-up by using the reference fold-decreases reported above (Scheme I).

#### Scheme I: Determination of the Percentage of Cleaved Stx2a after Toxin Purification

- Determine the IC_50_ of your Stx2a sample by the cell-free translation system described in the paper, in the presence and in the absence of DTT
- Calculate the fold decrease of your sample:

IC_50_ Stx2a/IC_50_ Stx2a + DTT = fold decrease

- Refer to the following fold decreases calculated from the IC_50_ values in Table 1:

IC_50_ Stx2a/IC_50_ Stx2a + DTT = 2.4 fold decrease; assuming A1 = 0%

IC_50_ cleaved Stx2a/IC_50_ cleaved Stx2a + DTT = 41.9 fold decrease; assuming A1 = 100%

- If the fold decrease calculated with your sample is ≤2.4 A1 = 0%
- If the fold decrease calculated with your sample is >2.4 and <41.9 use the following Equation

$$\frac{100\;\times \;\mathrm{fold}\;\mathrm{decrease}}{41.9}=\%\;A1$$

- If the fold decrease calculated with your sample is ≥41.9 A1 = 100%

A recent paper reported the comparison between a partially cleaved Stx2a sample purified by us in Austria (D.O.-H. and R.W., Medical University of Innsbruck, Austria) and the uncleaved toxin purified in Italy (D.C., E.P. and M.B., University of Bologna, Italy) [12]. Here, we have challenged our system with the Austrian toxin, in the absence and in the presence of DTT, showing a 34.2-fold decrease in the IC_50_ on protein synthesis, which is consistent with an increasing activity of the toxin upon reduction. The percentage of cleavage calculated by Scheme I (81.6% A1 fragment) is very similar to that obtained by SDS-PAGE under denaturing conditions, followed by densitometric analysis of the intensity of the A chain-derived protein bands (84% A1 fragment), as reported in [12].

### 2.4. Blocking Naturally Occurring Inhibitors of Cell-Free Protein Synthesis Present in Human Serum

**Figure 3:**
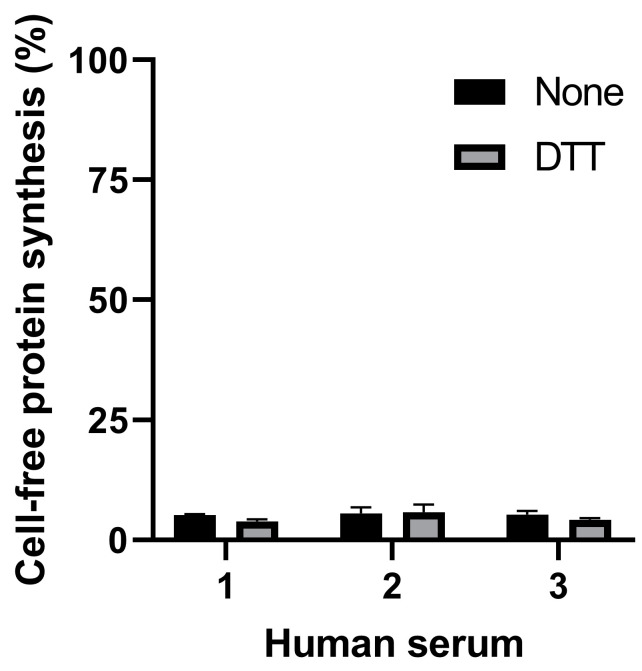
Effect of serum samples from three different healthy donors on cell-free protein synthesis in the presence or in the absence of DTT.

The present study is aiming at detecting the cleaved form of Stx2a in human serum. The presence of human serum from healthy donors (2 µL in the final 22 µL reaction) during the 3 µL preincubation step strongly impaired translation both in the presence and in the absence of DTT (Figure 3).

**Figure 4:**
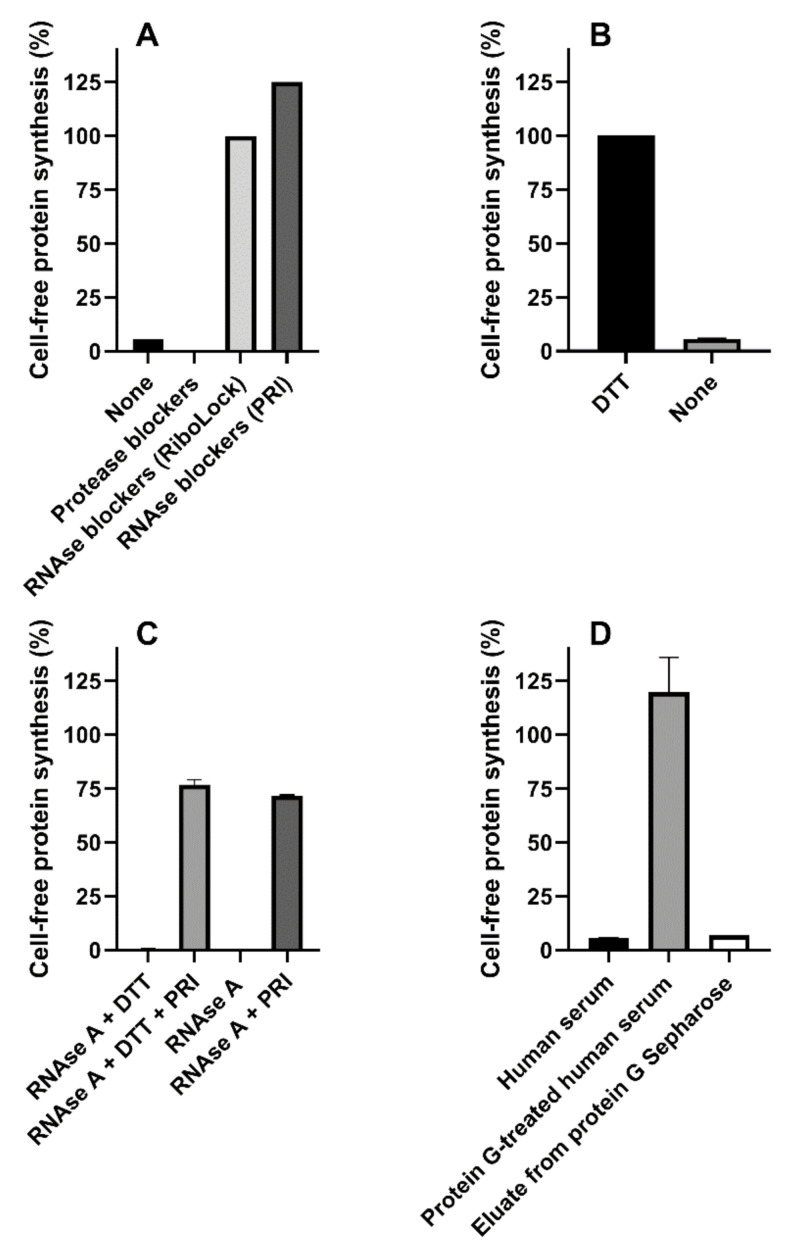
Mixed sera from three healthy donors or RNAse A were added to the cell-free translation system. (**A**) Mixed sera was assayed under reducing conditions in the absence or in the presence of protease (1x cOmpleteTM) or RNAse (20 U of RiboLock or Placental RNAse Inhibitor (PRI)) blockers; (**B**) Mixed sera was assayed in the presence of PRI under reducing and non-reducing conditions (*p* < 0.0001); (**C**) RNAse A (10 ng) was assayed giving strong inhibitory effects, the significant protective action of PRI under reducing and non-reducing conditions is shown (*p* < 0.0001); (**D**) Mixed sera were treated with protein G Sepharose and assayed. Comparison with untreated serum and protein G Sepharose eluate was performed.

Dialysis (6000–8000 molecular sieve) or gel-filtration (Microspin G-25 columns) of human serum did not change its effect on protein synthesis, suggesting the presence of high-molecular mass inhibitor/s. The effect of protease or RNAse blockers added to the reducing human serum-challenged system is shown in Figure 4A. Serin-, cysteine- and metal-proteases blockers (1x cOmpleteTM, Sigma-Aldrich, St. Louis, MO, USA) were ineffective, whereas the presence of blockers of RNAse A, B and C (20 U RiboLock, Thermo Scientific, Waltham, MA, USA) strongly protected the system from serum-induced inhibitory effects (Figure 4A), although partially affecting control protein synthesis (59% inhibition). A similar RNAse blocker (20 U Placental RNAse Inhibitor (PRI), Life Technology, Carlsbad, CA, USA) gave protection without affecting translation (Figure 4A); therefore, it was used in the experiments described throughout the paper.

Surprisingly, lack of DTT in the system restored the inhibitory activity by human serum even in the presence of PRI (Figure 4B). Optimal conditions for PRI activity require the presence of mild reducing conditions (1 mM DTT), and this might explain the results obtained in the absence of DTT. However, the data depicted in Figure 4C clearly demonstrate that, in our translation system, PRI fully blocked RNAse A from bovine pancreas both in the presence and in the absence of DTT. Thus, human sera contained different inhibitors of cell-free protein synthesis, i.e., an RNAse easily controlled by PRI, and a second translation inhibitor whose activity was affected by high DTT concentrations. Pretreatment of human serum mixtures with immobilized protein G (protein G Sepharose) restored protein synthesis activity under non-reducing conditions, hence suggesting the involvement of an immunoglobulin as a DTT-sensitive translation inhibitor. Re-addition to the system of the fraction eluted from protein G Sepharose gave inhibitory effects similar to those obtained with whole human serum, confirming our hypothesis, namely that an antibody-like molecule is responsible for the inhibitory effects (Figure 4D).

### 2.5. Detection of the Cleaved Form of Stx2a Added to Human Serum by the Cell-Free Translation System

**Figure 5:**
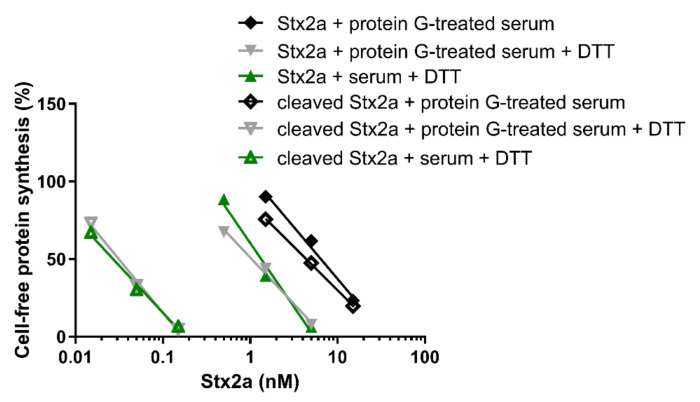
Effect of Stx2a on cell-free protein synthesis under reducing and non-reducing conditions in the presence of mixed sera from three healthy donors and PRI. The IC_50_ values calculated from the straight lines depicted above are reported in Table 2.

The cleaved and uncleaved forms of Stx2a were separately added to a mixture of human sera treated with protein G Sepharose (protein G treated-serum) and the experiments described in Figure 1 were repeated in the presence of PRI. Under non-reducing conditions, protein G treated-serum did not reduce the IC_50_ of both Stx2a forms (Figure 5, Table 1 and Table 2). In the presence of DTT and human serum, the inhibitory power of uncleaved toxin only slightly changed, while that of the cleaved form was greatly enhanced (Figure 5, Table 2). To demonstrate that pretreatment of sera with immobilized protein G had not removed other blood components affecting toxin activity, the same experiments were performed with untreated sera under reducing conditions, giving very similar results (Figure 5, Table 2).

**Figure 6:**
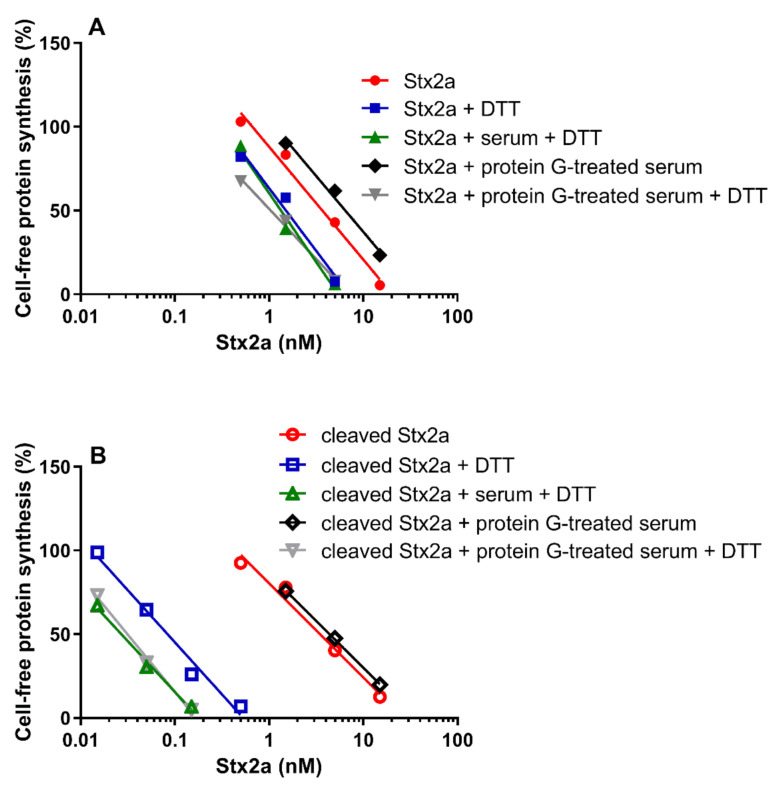
Effect of Stx2a on cell-free protein synthesis under reducing and non-reducing conditions. The experiments were performed in the presence of mixed sera from three healthy donors (untreated or treated with protein G Sepharose). When serum was present, PRI was added. (**A**) Uncleaved form of Stx2a; (**B**) Cleaved form of Stx2a. The IC_50_ values calculated from the straight lines depicted above are reported in Table 1 and Table 2. Mathematical and statistical details are reported in Supplementary Materials Table S1.

Data already shown in Figure 1 and Figure 5 were differently arranged in Figure 6, which groups in two different panels the straight lines obtained with uncleaved (panel A) and cleaved Stx2a (panel B). It is notable that the activity of uncleaved Stx2a slightly changes under the different assayed conditions (Figure 6A). Conversely, preincubation with DTT is the unique condition boosting the activity of cleaved toxin (Figure 6B), as exemplified by the extremely significant difference (~140 fold) in the IC_50_ obtained with trypsin-treated Stx2a in the presence of protein G treated-serum under non-reducing and reducing conditions (y-intercept difference, *p* = 0.0007).

Considering the 11-fold dilution in the assay (2 µL human serum in 22 µL final volume), toxin detection according to the equations reported in Supplementary Materials Table S1 (translation inhibitions between 10% to 100%) was possible at nearly µg/mL concentrations for uncleaved Stx2a, uncleaved Stx2a with DTT, and cleaved Stx2a (exact values in Scheme II). Conversely, cleaved Stx2a with DTT was detected by the herein described method in the range 6–126 ng/mL.

Serum Stx2a concentrations have been measured in STEC-infected patients before or after eHUS development. In a recent paper [4], we detected, using ELISA, concentrations of the toxin ranging from 2 to 6 ng/mL in patients developing eHUS. Therefore, only cleaved Stx2a could be detected in human serum by performing the assay under reducing conditions, although at the lower limit of detection (10%) with the highest concentration found in patients’ sera (6 ng/mL).

### 2.6. Lowering the Detection Limit of the Cell-Free Translation System for Cleaved Stx2a Added to Human Serum

To allow the detection of serum Stx2a at concentrations lower than 6 ng/mL, a further step was introduced. Protein G-treated human serum (100 μL) containing cleaved Stx2a (1.8, 3.6 or 6 ng/mL) was 5-fold concentrated by centrifugation on Centricon 30 (1 h at 12,000× *g*). After this treatment, cleaved Stx2a was detectable by our method in the presence of DTT. The obtained inhibitions were plotted on the equation of the appropriate straight line (Stx2a + protein G treated-serum + DTT), giving consistent results (2.3, 3.9 or 5.6 ng/mL, respectively). Concentrated human serum did not induce any effect on translation, although partially inhibiting protein synthesis in the absence of DTT. Therefore, we advise the measurement of Stx2a concentrations in patients’ serum by ELISA [17], for a comparison to be made between the obtained value (ng/mL) with the range of concentrations detectable by our method (Scheme II). If no inhibition is predicted, the serum sample should be assayed in the presence and in the absence of DTT to confirm this prediction and rule out the presence of unspecific Stx2a-unrelated effects on protein synthesis. After this preliminary experiment, the 5-fold concentrated serum sample can be assayed only in reducing conditions and the amount of cleaved Stx2a determined as described above according to Scheme II.

#### Scheme II: Detection of the Cleaved Form of Stx2a in Human Sera from STEC-Infected Patients

- Determine the concentration of Stx2 (ng/mL) present in human sera by ELISA [17] referring to a standard curve obtained by adding purified Stx2a to normal human serum at 2, 4, 6, 8 and 10 ng/mL and incubating 10 min at 37 °C. Serum samples (60 μL) for Stx2 detection for the standard curve should be treated with an equal volume of 0.4 M guanidinium chloride for 1 h at 37 °C, then 100 μL of the mixture should be assayed by using a commercially available kit (cat. n. 542010 Eurofins Abraxis, Warminster, PA, USA). The antibodies purchased in the kit recognize both the native and the cleaved form of the toxin.
- Refer to the following ranges of detectable Stx2a serum concentrations calculated from the equations of the straight lines obtained by the experiments described in the paper and reported in Supplementary Materials Table S1:

Stx2a + protein G-treated serum 1213–27,095 ng/mL (10–100% inhibition)

Stx2a + protein G-treated serum + DTT 167–5340 ng/mL (10–100% inhibition)

cleaved Stx2a + protein G-treated serum 632–25,754 ng/mL (10–100% inhibition)

cleaved Stx2a + protein G-treated serum + DTT 6–126 ng/mL (10–100% inhibition)

A. If inhibitions are predictable (≥10% ≤ 100%)

- Determine the effect on protein synthesis of the protein G-treated patient’s serum sample with respect to a pool of protein G-treated sera from healthy donors (at least 3) by the cell-free translation system described in the paper in the presence and in the absence of DTT.
- Calculate the percentage activity in each condition = %Act
- Use the b (y-intercept) and m (slope) of the appropriate straight line reported in Supplementary Materials Table S1 of the paper to calculate the serum concentration of the specific toxin form according to the following equation:
  $$10\frac{b-\%\mathrm{Act}}{-m}\times \;68\;\times \;11=\mathrm{ng}/\mathrm{mL}$$

It should be noted that concentrations akin to those found in patients’ sera are probably detectable only for cleaved Stx2 in reducing conditions.

B. If no inhibitions are predictable

- Confirm the absence of inhibition by determining the effect on protein synthesis of the protein G-treated patient’s serum sample with respect to a pool of protein G-treated sera from healthy donors (at least 3) by the cell-free translation system described in the paper in the presence and in the absence of DTT.
- If no inhibitions are detectable, concentrate the protein G-treated patient’s serum sample by 5-fold as described in the paper.
- Determine the effect on protein synthesis of the 5-fold concentrate protein G-treated patient’s serum sample with respect to a 5-fold concentrated pool of protein G-treated sera from healthy donors (at least 3) by the cell-free translation system described in the paper only in the presence of DTT.
- Calculate the percentage activity = %Act
- Use the b (y-intercept = −53) and m (slope = −68.49) of the straight line obtained with cleaved Stx2a + protein G-treated serum + DTT (Supplementary Materials Table S1) to calculate the serum concentration of cleaved Stx2a
  $$10\frac{-53-\%\mathrm{Act}}{68.49}\times \;68\;\times \;11=\mathrm{ng}/\mathrm{mL}$$

## 3. Discussion

In this paper, we employed a luminometric fractionated system [15] derived from the classic rabbit reticulocyte radioactive translating system, which resulted in higher sensitivity to whole untreated Stx2a with respect to the parent system. The lower amount of ribosomes in the derived system (0.125 pmoles vs 2.5 pmoles) would partially account for the higher sensitivity to these bacterial ribosome-inactivating toxins. Moreover, the requirement of a new synthesized fully active *Renilla* luciferase enzyme (luminometric system) to measure the translation rate with respect to the incorporation of a radioactive amino acid in the nascent protein (classic system) would amplify the consequences of the damaging effects on ribosomes.

The presence of the cleaved form of Stx2a was demonstrated by the strong increase of anti-ribosomal activity when the trypsin-cleaved form of the toxin was preincubated with optimal DTT concentrations (80 mM, 11 mM final concentration in the assay). However, the presence of human serum strongly interfered with cell-free protein synthesis because of the presence of a PRI-controlled RNAse and a DTT-sensitive high-molecular-mass component cleared by pre-incubation of sera with immobilized protein G, hence suggesting the involvement of immunoglobulins. It is well known that integrity and function of antibodies depend on the presence of disulphide bonds connecting their chains that could be broken by high DTT concentrations. On the other hand, RNAses could inhibit protein synthesis by affecting the integrity of ribosomal RNA, mRNA or transfer RNA during the assay. The presence of a translation-blocking antibody in normal human sera is attractive and is under scrutiny in our laboratory. It should be noted that substitution of rabbit ribosomes for human ribosomes did not change the inhibitory pattern.

Stx2a has been found to be associated with circulating cells in STEC-infected patients who developed eHUS and in those who recovered [4], while cell-free Stx2a fleetingly appearing in blood is considered the most ominous circulating form of the toxin [4,5,18]. The proposed method might be useful for the detection of cell-free Stx2a which circulate in human blood. Free Stx2a was found in sera from STEC-infected patients before the onset of eHUS [4,17,18] and its cleaved form could be easily detected by this method. The other form of cell-free Stx2a found in patients’s sera is particulate toxin (i.e., associated to extracellular vesicles), which is strictly related to the development of eHUS [4]. In this setting, Stx2a can be associated via B chains to Gb3Cer receptors present on the membranes of extracellular vesicles deriving from platelets and monocytes and/or via A chain to TLR4 present on the surface of the same cell-derived vesicles and in those from neutrophils [1,2,19]. The cleaved form of Stx2a present on the surface of extracellular vesicles is detectable only after reduction and release of the A1 fragment from the membrane. This seems to be possible for Gb3Cer-bound cleaved Stx2a, which consequently could be detected by this method. Conversely, TLR4-bound Stx2a is obviously uncleaved since the cleaved toxin does not bind to this receptor [12].

ELISA [17] and the herein proposed method detect Stx2 present in human serum at similar concentrations. ELISA is recommended if a rapid quantitative determination of Stx2 in patients’ blood is required for the diagnosis of STEC infections or for studies on the time-course of blood Stxs. Since, however, this ELISA [17] does not discriminate between uncleaved and cleaved Stx2, the detection of the latter form is possible only by applying the new method. In studies on the pathogenesis of eHUS, the advantage of applying both methods is twofold: to know the total amount of Stx2 in patients’ blood, and consequently, the proportion of cleaved and uncleaved toxins, and to avoid false positive data resulting from nonspecific inhibition of protein synthesis by patients’ sera. The application of this method to sera from STEC-infected patients during the early phase of the infection would clarify the role of A-cleaved Stx2a as a pathogenic factor in the onset of eHUS in children.

## 4. Materials and Methods

### 4.1. Materials and Toxin

Stx2a produced by *E. coli* C600 (933W) was purified by receptor analog affinity chromatography on (Galα1-4Galβ-O-spacer)-BSASepharose 4B (Glycorex, Lund, Sweden) [20], followed by a passage through ActiCleanEtox columns (SterogeneBioseparations, Carlsbad, CA, USA) to remove trace endotoxin contaminant. Purified Stx2a was stored at −80 °C in small aliquots and diluted before each assay with phosphate-buffered saline (PBS) containing 1% bovine serum albumin (BSA) or with human serum. A cocktail of protease blockers (cOmplete^TM^) was purchased by Sigma-Aldrich (St. Louis, MO, USA); blockers of RNAse A, B and C (RiboLock RNase Inhibitor and Placental RNase Inhibitor, PRI) were obtained by Thermo Scientific (Rozzano, Milan, Italy) and Life Technology (Monza, MB, Italy), respectively. Protein G Sepharose fast flow P3296, Microspin G-25 columns, Centricon 30 ultra 0.5 mL centrifugation filters were purchased by Merck Life Science (Milan, Italy). Human sera were collected from 7 healthy donors after obtaining informed consent and frozen at −20 °C. In most of the experiments, a pool of three different sera was used. Percentages of protein synthesis obtained in the presence of Stx2a and used for the determination of the IC_50_ values (Figures and Tables) were calculated with respect to controls run with pooled human sera or PBS-BSA in the absence of the toxin.

### 4.2. Cleavage of Stx2a

Stx2a (4 μg) was incubated [12] with 50 ng of trypsin (1 mg/mL in 0.1 mM HCl, diluted to 0.05 ng/mL with PBS) in 10 μL PBS pH 7.5 for 1 h at 37 °C. Then, 0.7 ng of the trypsin inhibitor PMSF (1 mg/mL in absolute ethanol diluted to 0.7 μg/mL with water) was added for 10 min at 37 °C. Native and cleaved Stx2a were analysed by SDS-PAGE under denaturing conditions, followed by densitometric analysis of the intensity of the A chain-derived protein bands, showing no or 96% A1 fragment, respectively.

### 4.3. Cell-Free Protein Synthesis Systems

The preparation of rabbit reticulocyte lysate and of the related fractions (post-ribosomal supernatant S-140 and ribosomal salt-wash) as well as of the protein synthesis Master Mix has been previously described in a dedicated method paper [15]. Human and rabbit ribosomes were prepared as previously described [15] from Raji cells cultivated as in [21] or from the reticulocyte lysate, respectively. The luminometric translation system reaction mixture described in [15] contained the following components in a final volume of 22 µL: 3 µL preincubation mixture (containing PBS-BSA, or PBS-BSA + DTT or human serum or human serum + DTT or Stx2a or Stx2a + DTT as detailed in text and figures), 4.4 µL Master Mix 5X (containing 150 mM HEPES/KOH, pH 7.5, 400 mM KCl, 5 mM magnesium acetate, 250 µM of each amino acid, 10 mM ATP, 1.25 mM GTP, 25 mM creatine phosphate, 0.9 mg/mL creatine phosphokinase, 2.5 mM DTT, 2 mM spermidine), 8.8 µL S-140, 0.9 µL ribosomal salt wash, 1 µL capped mRNA encoding *Renilla* luciferase (0.3 µg, transcribed from pRL-FL plasmid), 0.5 µL PRI (20 U, only when human serum was added) and 2 µL human or rabbit ribosomes (0.125 pmol in 10 mM Tris HCl, pH 7.5, 2 mM magnesium acetate and 100 mM ammonium acetate), water to the final volume. After 60 min at 30 °C, the synthesis was stopped on ice and the luciferase activity was measured using a Dual-Luciferase Reporter assay (Promega, Milan, Italy) following the manufacturer’s instructions.

### 4.4. Treatment of Human Serum with Protein G Sepharose

The commercial suspension protein G Sepharose was diluted with an equal volume of 20% ethanol. A 375 µL aliquot was centrifuged for 4 min at 200× *g* at 4 °C in Eppendorf tubes. The resin was washed three times with two volumes of PBS followed by centrifugation as above. Human serum samples (125 µL) were added to the resin and incubated for 15 min at room temperature (24 °C) in a rotating mixer. Then, the mixture was centrifuged as above and 100 µL serum was withdrawn (protein G-treated serum). Bound antibodies were eluted by adding 500 µL of 100 mM glycine/HCl, pH 2.7. The eluate was immediately back-titrated to neutral pH by adding 10 µL of 1 M KOH and dialyzed 4 h against PBS (1 L). The final eluate was used in preliminary experiments to demonstrate the capture of the translation inhibitor/s present in human sera. Stx2a was added to human serum samples before or after protein G Sepharose treatment, as indicated in the text.

### 4.5. Statistics

Data analysis was performed with GraphPad Prism 8. IC_50_ values were calculated by the least-squares method applied to the linear regression between percentage activity and log of inhibitor concentrations. Differences in continuous variables were tested with a *t*-test, a value of *p* < 0.05 was considered statistically significant. Correlation between variables was assessed by the Pearson correlation coefficient. Differences in slopes and elevations of the different straight lines were tested by linear regression. No significant differences have been observed in slopes, the significant differences in elevations are reported in the text.

## Figures and Tables

**Table 1:** IC_50_ of Stx2a assayed in the cell-free translation system.

Additions	IC_50_ (nM)
Stx2a	3.680
Stx2a + DTT	1.511
cleaved Stx2a	3.475
cleaved Stx2a + DTT	0.083

**Table 2:** IC_50_ of Stx2a added to human serum and assayed in the cell-free translation system.

Additions	IC_50_ (nM)
Stx2a + protein G-treated serum	6.449
Stx2a + protein G-treated serum + DTT	1.042
Stx2a + serum + DTT	1.339
cleaved Stx2a + protein G-treated serum	4.389
cleaved Stx2a + protein G-treated serum + DTT	0.031
cleaved Stx2a + serum + DTT	0.027

## Data Availability

The data presented in this study are available on request from the corresponding author.

## Supplementary Materials

The following are available online at https://www.mdpi.com/2072-6651/13/2/94/s1, Table S1: IC_50_ of Stx2a on the rabbit reticulocyte-derived fractionated cell-free translation system: Equations and statistical analysis of the straight lines.
