# Supplementary material for: Method for the Detection of the Cleaved Form of Shiga Toxin 2a Added to Normal Human Serum
Source: Toxins (Basel). 2021 Jan 26;13(2):94. doi: 10.3390/toxins13020094 (PMC7911550; doi:10.3390/toxins13020094)
Supplement: Supplementary file 1 [file toxins-13-00094-s001.pdf]

Supplementary Materials: Method for the Detection of the Cleaved Form of Shiga Toxin 2a Added to Normal Human Serum

Lucrezia Rocchetti, Beatrice Munari, Elisa Varrone, Elisa Porcellini, Dorothea Orth-Höller, Reinhard Würzner, Domenica Carnicelli and Maurizio Brigotti

Table 1. IC_{50} of Stx2a on the rabbit reticulocyte-derived fractionated cell-free translation system: equations and statistical analysis of the straight lines.

Additions	IC_{50} (nM)	r^2	Equation
Stx2a	3.680	0.9834	$y = -67.31x + 88.09$
Stx2a + DTT	1.511	0.9714	$y = -74.79x + 63.41$
Stx2a + protein G-treated serum	6.449	0.9878	$y = -66.71x + 104.0$
Stx2a + protein G-treated serum + DTT	1.042	0.9918	$y = -59.81x + 51.06$
Stx2a + serum + DTT	1.339	0.9798	$y = -82.14x + 60.42$
cleaved Stx2a	3.475	0.9796	$y = -56.20x + 80.41$
cleaved Stx2a + DTT	0.083	0.9804	$y = -62.21x - 17.06$
cleaved Stx2a + protein G-treated serum	4.389	0.9994	$y = -55.89x + 85.90$
cleaved Stx2a + protein G-treated serum + DTT	0.031	0.9944	$y = -68.49x - 53.00$
cleaved Stx2a + serum + DTT	0.027	0.9902	$y = -60.66x - 45.13$
